# Basophil activation test: food challenge in a test tube or specialist research tool?

**DOI:** 10.1186/s13601-016-0098-7

**Published:** 2016-03-15

**Authors:** Alexandra F. Santos, Gideon Lack

**Affiliations:** Department of Paediatric Allergy, Division of Asthma, Allergy and Lung Biology, King’s College London, London, UK; MRC and Asthma UK Centre in Allergic Mechanisms of Asthma, London, UK

**Keywords:** Basophil activation test, CD63, CD203c, Food allergy, Diagnosis, Immunotherapy, Peanut allergy

## Abstract

Oral food challenge (OFC) is the gold-standard to diagnose food allergy; however, it is a labour and resource-intensive procedure with the risk of causing an acute allergic reaction, which is potentially severe. Therefore, OFC are reserved for cases where the clinical history and the results of skin prick test and/or specific IgE do not confirm or exclude the diagnosis of food allergy. This is a significant proportion of patients seen in Allergy clinics and results in a high demand for OFC. The basophil activation test (BAT) has emerged as a new diagnostic test for food allergy. With high diagnostic accuracy, it can be particularly helpful in the cases where skin prick test and specific IgE are equivocal and may allow reducing the need for OFC. BAT has high specificity, which confers a high degree of certainty in confirming the diagnosis of food allergy and allows deferring the performance of OFC in patients with a positive BAT. The diagnostic utility of BAT is allergen-specific and needs to be validated for different allergens and in specific patient populations. Standardisation of the laboratory methodology and of the data analyses would help to enable a wider clinical application of BAT.

## Background

The gold-standard for the diagnosis of food allergy is oral food challenge (OFC) [1]. However, OFC requires the ingestion of the suspected culprit food and can cause an acute allergic reaction, which is potentially severe [2]. For this reason, OFC need to be performed in a supervised environment with the facilities and expertise to treat allergic reactions and anaphylaxis, should they occur. OFC can cause significant anxiety in patients, parents and even clinical staff, as it involves considerable risk. Thus, whenever possible, the diagnosis of food allergy is established by a recent convincing history of an IgE-mediated allergic reaction to the culprit food combined with evidence of IgE sensitization to the same food by skin prick test (SPT) and/or serum specific IgE (sIgE) [3]. OFC are reserved for the cases where the results of SPT and/or sIgE are equivocal. With increased awareness and increased prevalence of food allergy and food sensitization, more and more patients are being tested for food allergy. The absence of a history of oral exposure to allergenic foods, either resulting in an allergic reaction or in the absence of clinical symptoms, can make the interpretation of the results of the SPT and sIgE particularly challenging. Infants and young children who have never had certain allergenic foods constitute a considerable proportion of patients seen in Allergy clinics and often need an OFC to clarify their allergic status. This results in increasing demand in the performance of OFC. Allergy services have difficulty in responding to this demand and patients may need to wait several months before being offered an exact diagnosis of food allergy or food tolerance by OFC, which can lead to unnecessary dietary restrictions and to significant anxiety associated with diagnostic uncertainty. OFC is also the gold-standard to assess resolution of food allergy, to determine the threshold dose and to monitor the clinical response to immunomodulatory treatments for food allergy. In research studies, allergic patients often have to undergo repeated OFC to assess whether there has been any clinical improvement.

The BAT, being a functional assay, has the potential to resemble more closely the clinical phenotype of patients than allergy tests that merely detect the presence of allergen-specific IgE. In simple terms, the BAT can be seen as an OFC in a test tube, where instead of giving the food to a child by mouth, basophils involved in acute allergic reactions are exposed to a food extract in a test tube. Despite the analogy, differences between the two procedures and their clinical applications can be pointed out and here lays the question as to whether BAT can loyally mimic the gold standard OFC, i.e. whether BAT can reproduce in vitro the allergic reaction that happens in vivo during a positive OFC.

### The basophil activation test

**Fig. 1:**
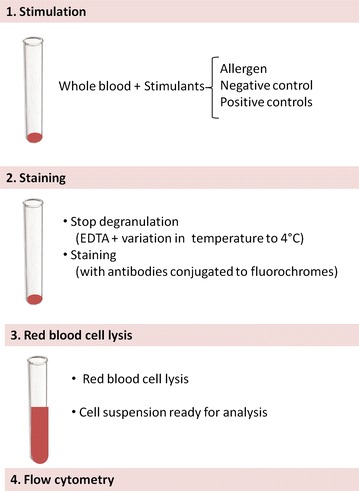
Diagram of the laboratory procedure for the basophil activation test. Following stimulation of blood cells with allergen or controls, blood cells are stained with antibodies coupled to a fluorochrome, which allow the identification of cells and the measurement of the expression of activation markers using a flow cytometer

The BAT is a flow cytometry-based assay where the expression of activation markers is measured on the surface of basophils following stimulation with allergen [4, 5]—Fig. 1. A positive basophil activation test can be seen as an in vitro surrogate of an acute allergic reaction in vivo. In a study of patients allergic to hymenoptera venom, up-regulation of basophil activation markers was observed both in vitro following stimulation with yellow jacket or honey bee venom and ex vivo following a positive sting challenge [6]. In the same study, there was a general agreement between the clinical presentation (systemic reaction versus large local reaction) and the results of BAT, suggesting that the BAT is a potential biomarker of anaphylaxis. Also in food allergic patients a good agreement was found between the results of BAT and the outcome of OFC. In patients allergic to alpha-gal with delayed immediate-type allergic reactions to red meat, the activation of basophils ex vivo in blood collected at different time points coincided with the development of systemic allergic reactions in vivo during the OFC [7]. The results of this study reinforce the role of basophils in food-induced IgE-mediated allergic reactions and anaphylaxis.

Different cell-surface markers can be used to identify basophils in whole blood, including IgE, CD123 (with HLA-DR), CCR3 or CRTH2 (with CD3) or CD203c [4].

In peripheral blood, IgE is detected on basophils, dendritic cells, eosinophils, monocytes, macrophages, B cells and platelets, thus it is not specific for basophils. CD123 is the low affinity (α) subunit of the IL-3 receptor. It is expressed in high levels on plasmacytoid dendritic cells and basophils and in low levels on monocytes, eosinophils, myeloid dendritic cells and subsets of haematologic progenitor cells. Additional staining with HLA-DR discriminates between HLA-DR-negative basophils and HLA-DR-positive dendritic cells and monocytes. CCR3 is the receptor for C–C type chemokines (e.g. eotaxin, MCP and RANTES) and is highly expressed on basophils and eosinophils but also on Th1 and Th2 cells. CRTH2 is another marker that is expressed by basophils, eosinophils and T cells, and thus like CCR3, requires a T cell marker, such as CD3, to distinguish basophils from T cells. CD203c is constitutively and specifically expressed on basophils and therefore can be used as a single identification marker or in combination with other markers.

Following stimulation with allergen, the expression of different proteins is up-regulated on the surface of basophils [4], namely CD63 [8] and CD203c [9, 10]. CD63 is a lysosomal-associated membrane protein (LAMP), which is not expressed on the surface of resting basophils but only on the membrane of the granules inside the cells [8]. When the granules fuse with the plasmatic membrane of the basophils during degranulation, CD63 becomes expressed on the surface of basophils [10]. CD203c is an enzyme that cleaves phosphodiester and phosphosulphate bonds, hydrolytically removing 5′-nucleotides successively from the 3′-hydroxy-termini of oligonucleotides. It is exclusively and constitutively expressed in low levels on the surface of basophils and mast cells and its expression increases with cell activation. Basophil activation markers seem to form two distinct groups of markers that are up-regulated concomitantly: one including CD63, CD107a and CD107b and another including CD203c, CD13 and CD164 [11]. CD63 and CD203c are the most commonly used basophil activation markers.

The laboratory procedure of the BAT consists of three stages: cell stimulation, cell staining and flow cytometry—Fig. 1. Blood should be processed as soon as possible after blood collection, as basophils lose their viability and reactivity over time. However, studies have been performed with samples stored at +4 °C up to 24 h [12]. A small volume of blood (c.a. 1–2 ml depending on the number of conditions) is required for BAT. Crude allergen extracts or purified or recombinant allergens can be used for cell stimulation. Different allergen concentrations should be used, as the sensitivity of the basophils to specific allergen stimulation varies among patients.

**Fig. 2:**
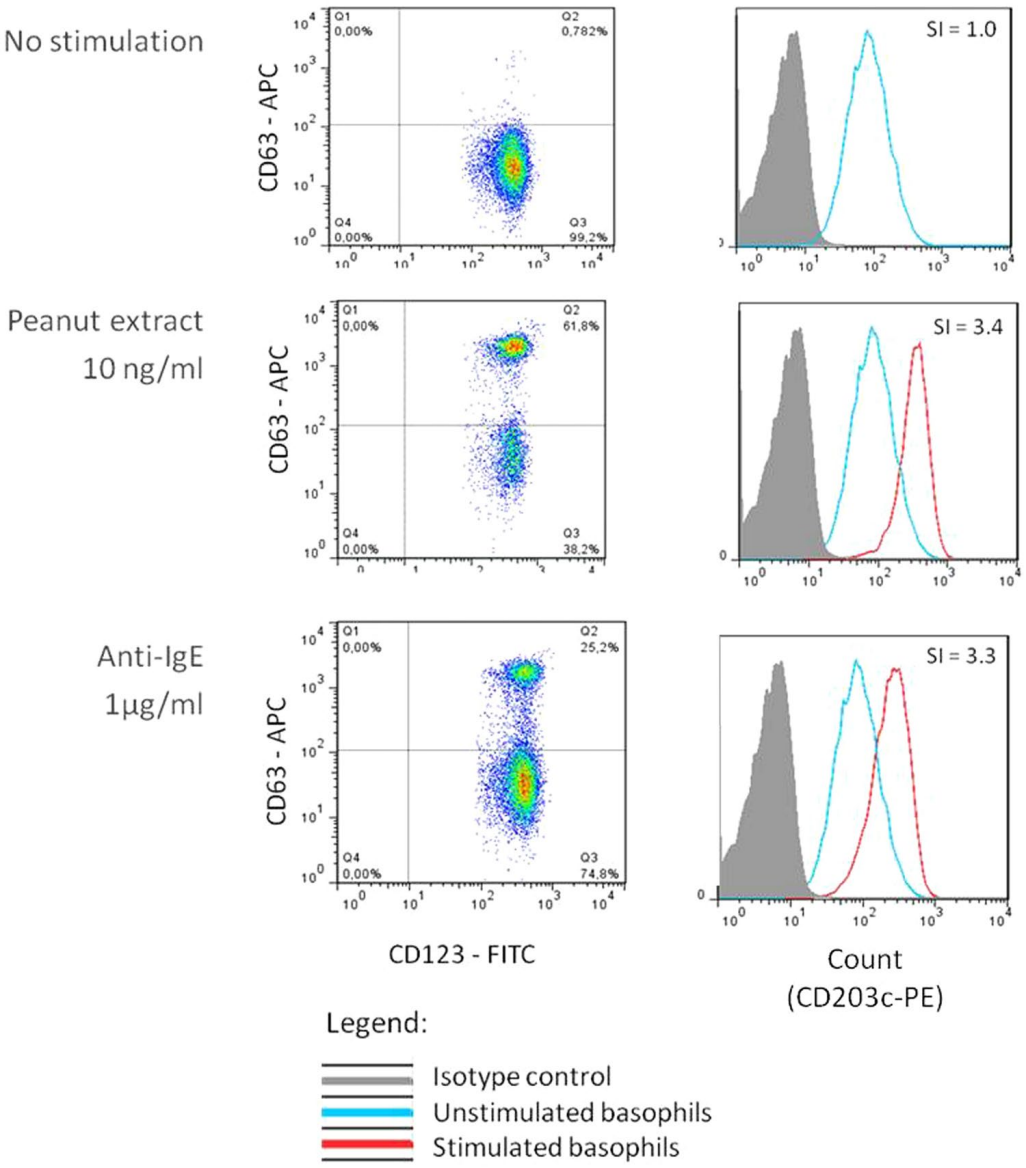
*Dot plots* and histograms showing the expression of CD63 and CD203c on the surface of basophils in different conditions. Unstimulated cells (negative control) and cells stimulated with peanut or with anti-IgE (positive control) are represented. The expression of CD63 is measured as the percentage of positive basophils (*left panel*) and the expression of CD203c is measured as the stimulation index (SI), i.e. the ratio of the mean fluorescence intensity of stimulated cells and the negative control (*right panel*)

The results of BAT can be determined in terms of percentage of basophils expressing the defined activation marker or in terms of mean fluorescence intensity (MFI) by calculating the stimulation index, i.e. the ratio between the MFI of the selected condition and the MFI of the negative control. The former is usually used for CD63 as CD63 is not expressed in resting cells and its expression after activation is bimodal. The latter is usually used for CD203c which is already expressed in resting cells and its increase following allergen stimulation is unimodal—Fig. 2.

**Fig. 3:**
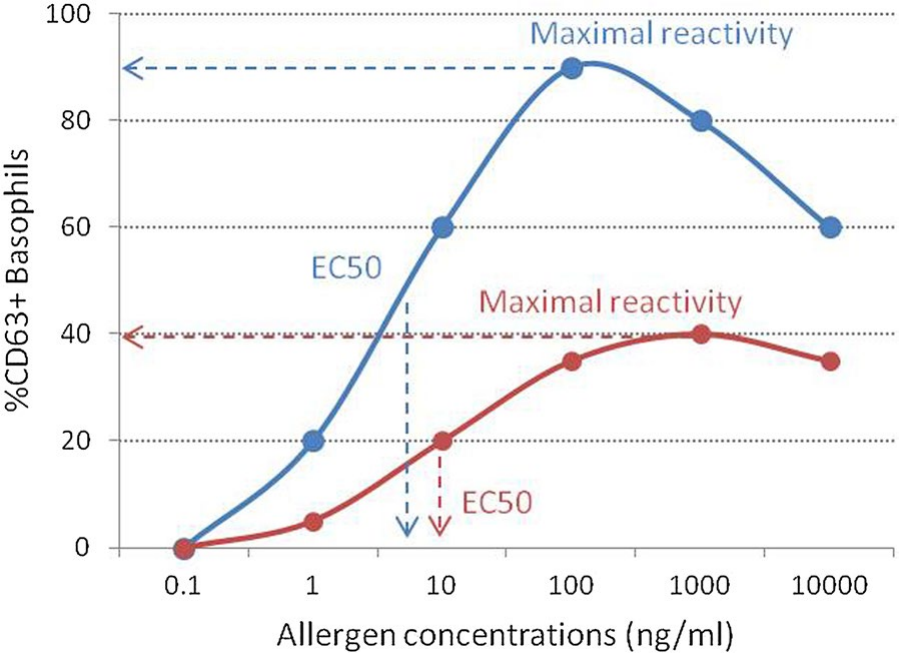
Basophil reactivity and basophil sensitivity. Two examples of dose–response curves of basophil activation following stimulation with various concentrations of allergen from two different patients are represented. The proportion of CD63+ positive cells is a measure of basophil reactivity and EC_50_, the effective concentration at 50 % of the maximal activation, is a measure of basophil sensitivity

In allergic patients, allergen-induced basophil activation typically results in a bell-shaped dose–response curve, with increasing concentrations of the allergen (usually 5–6 log difference) leading to a progressive increase in the expression of the basophil activation markers until reaching a plateau—Fig. 3.

There is a large degree of variability in the basophil response to allergen between individuals. In order to express this heterogeneity and to compare basophil responses between different patients, various parameters can be determined based on the dose–response curve, such as CD-max and EC_50_ (50 % effective concentration) or CD_sens_. CD-max is the maximal activation and corresponds to the maximum proportion of activated basophils at any concentration of allergen [5]. EC_50_ is the effective dose at 50 % of the maximal activation, and can also be represented as CD_sens_. First described by Johansson [13], CD_sens_ is the inverse of the half-maximal effective concentration, i.e. the concentration at which basophil activation is half of the maximum activation, times 100 and can be calculated using the formula: CD_sens_ = 1/EC_50_ × 100. CD_max_ and CD_sens_ are measures of basophil reactivity and of basophil sensitivity, respectively. Basophil reactivity can be defined as the degree of basophil activation, i.e. the proportion of activated basophils, and can also be measured as the percentage of CD63-positive basophils at different allergen concentrations or as the ratio of the percentage of CD63-positive after stimulation with allergen and with anti-IgE. Basophil sensitivity refers to the concentration of allergen at which basophils become activated and can be expressed as a percentage of the maximal effective dose (e.g. EC_5_, EC_10_) apart from EC_50_ and CD_sens_, previously mentioned. Figure 3 represents the basophil response of two different individuals, one with higher basophil reactivity and sensitivity (blue) and the other with lower basophil reactivity and sensitivity (red), i.e. with a smaller proportion of basophils becoming activated in response to higher concentrations of the allergen. Shreffler and Patil [14] have proposed a novel parameter to measure basophil responses, the area under the dose–response curve, which has the advantage of combining basophil reactivity and basophil sensitivity.

### Using the basophil activation test to diagnose food allergies

In a recently published study [15], we assessed the utility of the BAT to diagnose peanut allergy in a well-characterized population of peanut allergic, peanut sensitized and non-sensitized children. BAT showed high accuracy (97 %) in diagnosing peanut allergy and allowed a reduction in the number of OFC required by 66 %. We validated the diagnostic cut-offs in a prospectively and independently recruited population and the diagnostic performance of BAT was still very good in this second study population. Over the past few years, other studies have assessed the performance of BAT in the diagnosis of allergy to different foods, including peanut [12, 15–18], cow’s milk [19, 20], egg [17, 19], wheat [21–25], hazelnut [26–28], shellfish [29] and peach [30–32], as well as in the diagnosis of pollen-food syndromes [33–35]—Table 1. Case reports and small case series have suggested that BAT may also be useful to diagnose allergy to sesame [36] and to less common elicitors of IgE-mediated food allergic reactions, such as rice [37] and short chain galacto-oligosaccharides present in prebiotics [38]. A recently published position paper from the European Academy of Allergy and Clinical Immunology reviews the clinical applications of the BAT [39].

**Table 1:** Examples of study assessing the utility of BAT to diagnose food allergy

Food	Author year	N	Cut-offs	Sensitivity	Specificity
Peanut	Santos 2014 [15]	N = 104	≥4.78 % CD63+	97.6 %	96.0 %
		Validation population N = 65		83.3 %	100 %
	Glaumann 2012 [12]	N = 38	ND	92 %	77 %
	Javaloyes 2012 [16]	N = 26	ND	92 %	95 %
	Ocmant 2009 [17]	N = 75	≥9.1 % CD63+	87 %	94 %
Hazelnut	Brandström 2015 [28]	N = 40	CD-sens > 1.7	100 %	97 %
Egg	Ocmant 2009 [17]	N = 67	≥5 % CD63+	77 %	100 %
Cow’s milk	Sato 2010 [19]	N = 50	SI CD203c ≥ 1.9	89 %	83 %
Wheat	Tokuda 2009 [22]	N = 58	≥14.4 % CD203c+	85 %	77 %
Apple (PFS)	Ebo 2005 [34]	N = 61	Vs sensit. ≥17 % CD63+ Vs NA ≥10 %	Vs sensit. = 88 % Vs NA = 100 %	Vs sensit. = 75 % Vs NA = 100 %
Hazelnut (PFS)	Erdmann 2003 [33]	N = 30	≥6.7 % CD63+	85 %	80 %
Celery (PFS)			≥6.3 % CD63+	85 %	80 %
Carrot (PFS)			≥8.9 % CD63+	85 %	85 %

*N* number of study participants, *PFS* pollen-food syndrome, *ND* not determined, *Vs* versus, *Sensit.* sensitised but tolerant, *NA* non-sensitised non-allergic, *SI *stimulation index

Various factors may influence the diagnostic performance and cut-off values of BAT in different studies, some related to the study population, some related to the study design, some related to the laboratory procedure and to the methodology adopted for data analyses—Table 2. Existing studies are heterogeneous in most of these aspects, which limits their comparability and a wider application of the diagnostic cut-offs determined in specific studies. The criterion to diagnose each food allergy is allergen-specific and the diagnostic accuracy may not be the same for different allergens. Additionally, the cut-offs defined in one population are not necessarily directly transferrable to another population from a different geographical location assessed in a different Allergy centre. One limitation of BAT is the fact that a small proportion of patients tested have non-responder basophils (i.e. basophils that respond to a non-IgE-mediated positive control but not to IgE-mediated stimulants) and therefore have an uninterpretable result for the test. Additional challenges in translating the BAT from a research method to a diagnostic test in the clinic are related to the standardisation of the assay and its reproducibility and also to the cost-effectiveness of including BAT in the diagnostic approach of patients with suspected food allergy. These aspects have not yet been established and require further research.

**Table 2:** Examples of factors that can influence the diagnostic cut-offs for BAT in food allergy [4, 14, 39, 40, 41]

Study population	Prevalence of the food allergy in the population
	Origin of the study population (e.g. recruited from a specialized Allergy clinic or from the general population)
	Geographical location
	Associated respiratory and food allergies
Study design	Inclusion criteria (e.g. whether sensitized as well as non-sensitized patients were included in the study)
	Gold-standard used as a comparator to determine the diagnostic cut-offs
	Criteria for performing OFC (e.g. whether patients with a history of anaphylaxis or other risk factors for a severe reaction or with high levels of IgE or large wheals on skin prick test were included)
	OFC protocol (e.g. criteria for stopping the OFC, criteria for a positive OFC, intervals between doses and duration of OFC)
Laboratory procedure	Interval between blood collection and the performance of BAT
	Allergen extracts or purified/recombinant allergens used
	Concentration of the allergens
	Pre-incubation with IL-3
	Markers and antibodies (e.g. clones, fluorochromes) used to identify the basophils and to detect basophil activation
Flow cytometry data analyses	Adopted gating strategy
	Parameters used as the outcomes of the test [e.g. CD63 or CD203c, % or SI, CD-sens, area under the dose–response curve]
	Definition of negative gate
	Whether results were corrected for the background
	Cytometer used and application settings

**Fig. 4:**
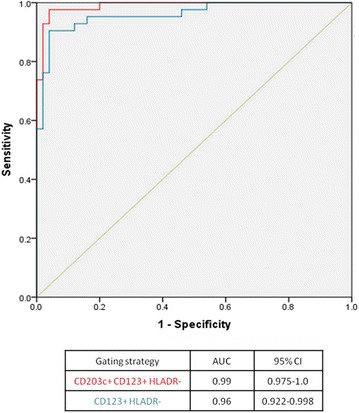
Enhancement of the diagnostic accuracy of the basophil activation test to peanut using the basophil gating strategy SSC^low^/CD203c+/CD123+/HLA-DR- (in *red*) compared to using SSC^low^/CD123+/HLA-DR- (in *blue*). ROC curves of the average  % CD63+ basophils at 10 and 100 ng/ml of peanut extract using the two different gating strategies

The methodology adopted to perform the laboratory procedure and to analyse the flow cytometry data can have a significant impact on the results obtained for the BAT and, consequently, in its diagnostic accuracy. For example, identifying basophils using an anti-IgE antibody can activate the cells and alter the results obtained with a different method to identify the basophils. The expression of certain basophil identification markers, such as CCR3 [42] and CD123 [43] can change following basophil activation. In a recent study, we described that in about a quarter of patients, the expression of CD123, as detected by flow cytometry, can be reduced following basophil activation and therefore lead to a significant loss-to-analyses of activated cells using methods that rely on this marker to identify basophils. This could result in an increased number of misdiagnosis, particularly false-negatives, with important consequences for individual patients. Adding the basophil specific marker CD203c to the gating strategy retained the cell number and allowed to reduce the number of false-negatives (from 5 to 1 %). The modified gating strategy improved both the sensitivity (from 88 to 98 %) and the specificity (from 94 to 96 %) of BAT, resulting in an overall enhancement of the accuracy of BAT to diagnose peanut allergy from 91 to 97 %—Fig. 4. In order to conduct and interpret BAT successfully, “the devil is in the detail”; therefore, it is very important to carefully consider the methodological aspects of BAT.

Overall, as a diagnostic test, BAT has shown high specificity and positive predictive value. We validated the diagnostic cut-offs determined for peanut allergy in an independent prospectively recruited population [15] and the specificity and positive predictive value of BAT reached 100 %. The high specificity is an important addition to existing allergy tests, such as SPT and sIgE, which have high sensitivity but are not very specific. The high specificity implies that a positive BAT confirms the diagnosis of food allergy with confidence but a negative BAT does not necessarily exclude the diagnosis. Depending on the cost-benefit ratio and safety aspects, OFC can be done in patients where BAT provides an inconclusive result (namely patients with non-responder basophils) or in patients where BAT provides an inconclusive result and in patients where BAT was negative.

**Fig. 5:**
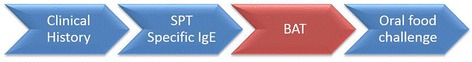
The basophil activation test can be useful in selected patients, in whom the combination of the clinical history and skin prick test (SPT) and/or specific IgE is inconclusive, before referring for oral food challenge. A positive BAT confirms the diagnosis of food allergy and allows to defer the performance of OFC. An equivocal or a negative BAT should be followed by OFC to clarify the diagnosis

The approach to decide about the need for OFC following BAT also depends on how the result of BAT is considered in the context of the results of other allergy tests, either in combination, when all the results available are considered simultaneously, or sequentially, where BAT is performed only in patients who had equivocal or discordant results for the other allergy tests. In our previously cited peanut study [15], we compared the performance of BAT with that of other allergy tests done in parallel. BAT performed better than SPT, specific IgE to peanut and specific IgE to Ara h 2 and other peanut components. Considering single tests, the most accurate diagnostic test was the BAT. In order to make the most of the information available, the results of BAT can be used in combination with the results of other tests [15, 17, 44, 45]. However, generally, the more tests used, the higher the diagnostic uncertainty (and the higher the number of OFC) given that different tests can provide contradictory results [15]. Better than combining the results of allergy tests simultaneously may be to use BAT sequentially in the food allergy diagnostic work-up, in patients who had an inconclusive result for the other allergy tests [15]. This approach can be advantageous also from a feasibility point of view, considering the practicalities involved in the performance of BAT, namely the need for fresh blood and the resources and technical expertise required. It would not be practical or even necessary to perform BAT in all the patients being investigated for suspected food allergy. BAT can be reserved for selected cases, particularly cases where there is no history of oral exposure to the food or the clinical history is unclear and the results of SPT and specific IgE are inconclusive [15]. BAT could be used as a second step in the diagnostic work-up, following clinical history and SPT and/or sIgE, in difficult cases, before deciding whether an OFC is required [15]—Fig. 5. The diagnostic accuracy and the superiority of BAT over skin prick test and specific IgE needs to be assessed with other allergens and in other clinical settings.

Apart from distinguishing food allergic and food tolerant patients, the results of BAT can provide additional information about the characteristics of food-induced reactions that may be helpful in the management of allergic patients [46, 47]. Different parameters of the BAT have been shown to reflect different characteristics of the allergic reactions, with the proportion of activated basophils (basophil reactivity) reflecting the severity of allergic symptoms and the dose at which basophils react to allergen in vitro (basophil sensitivity) reflecting the dose of food protein at which patients reacted during OFC [46]. These findings in peanut allergy have been reproduced in a subsequently published study [47] and may be applicable to other food allergies. In any case, the result of BAT should be taken in the context of other clinical features and risk factors for severity, when assessing food-allergic patients.

### Using the basophil activation test to monitor acquisition of tolerance to foods and the clinical response to immunomodulatory treatments for food allergy

Reflecting closely the clinical phenotype of allergic and tolerant patients, BAT can be useful in assessing the natural resolution of food allergies that are commonly outgrown over time, such as cow’s milk [44], egg [19] and wheat [45] allergies, and in determining when to re-challenge the patients to assess whether the food can be reintroduced in the diet. BAT has shown to distinguish different phenotypes of patients with cow’s milk and egg allergies, namely patients that tolerate extensively heated forms of these foods while still reacting to the unheated foods from patients who react to both extensively heated and unheated milk or egg [19, 48, 49].

BAT has also been used to monitor clinical response to immunomodulatory treatments for food allergy in research studies. Overall, in studies of immunotherapy to foods such as peanut [50–54], cow’s milk [55] and egg [56, 57], BAT has shown decreased basophil reactivity to the respective food allergens with treatment, which is particularly evident at the lower concentrations of the allergen, reflecting the decrease in basophil sensitivity. Interestingly, Thyagarajan et al. [52] showed that, during peanut OIT, the reduction in basophil activation was not only happening in response to peanut but also to the bystander egg allergen (in egg allergic patients) and to anti-IgE but not to the non-IgE-mediated positive control, fMLP (formyl-methionyl-leucyl-phenylalanine), suggesting that the pathway downstream the IgE receptor had become anergic. In a study of omalizumab in peanut allergic patients [58], CD203c expression in the BAT decreased during treatment and returned to pre-treatment levels after cessation of this therapy. Finally, the Chinese herbal medicine FAHF-2 [59] also showed a significant inhibitory effect in basophil response in patients with allergy to different foods in parallel with clinical improvement.

Taken together, these studies illustrate that BAT can be repeated in the same patients over time to assess the changes in the immune response to food allergens with some sort of intervention, being it oral immunotherapy, sublingual immunotherapy, omalizumab, or other immunomodulatory therapeutic or preventive strategies.

### Future perspectives

With the view of applying BAT to the diagnosis of food allergy in clinical practice, further research is needed to define and validate diagnostic cut-offs for specific allergens and in different patient populations. Standardization of the laboratory procedures would be important to allow the comparability of the results of BAT between centers. This would require standardization of the protocol for the in vitro assay and of the flow cytometry and data analyses’ methods. The use of similar methodology for BAT would allow to compare the results of BAT in different centers, both for clinical and for research purposes, including in multicenter studies.

Once appropriately validated for the diagnosis of specific food allergies, BAT can be used to monitor the clinical response to immunomodulatory treatments such as allergen-specific immunotherapy and biologicals. BAT also has an enormous potential for mechanistic studies to improve our understanding of the role of basophils in the immune mechanisms of food allergy and food tolerance.

## Conclusions

BAT is a valuable research tool and has shown promise as a clinically useful test. Recent studies have shown that BAT diagnoses food allergy with high accuracy and can be particularly useful in cases with unclear clinical history or equivocal results of other diagnostic tests, before deciding on whether oral food challenges are required. BAT can also be used to monitor the clinical response to immunomodulatory treatments for food allergy. Further studies to define and validate diagnostic cut-offs values, to standardize the adopted methodology and to assess its cost-effectiveness are desirable in order to enable a wider use of BAT in clinical practice.

## Abbreviations

BAT: basophil activation test
MFI: mean fluorescence intensity
OFC: oral food challenge
SI: stimulation index
sIgE: specific IgE
SPT: skin prick test

## References

[1] Boyce JA, Assa’ad A, Burks AW, Jones SM, Sampson HA, Wood RA, Plaut M, Cooper SF, Fenton MJ, Arshad SH (2010). Guidelines for the diagnosis and management of food allergy in the United States: report of the NIAID-sponsored expert panel. J Allergy Clin Immunol.

[2] Perry TT, Matsui EC, Conover-Walker MK, Wood RA (2004). Risk of oral food challenges. J Allergy Clin Immunol.

[3] Du Toit G, Santos A, Roberts G, Fox AT, Smith P, Lack G (2009). The diagnosis of IgE-mediated food allergy in childhood. Pediatr Allergy Immunol.

[4] Ebo DG, Bridts CH, Hagendorens MM, Aerts NE, De Clerck LS, Stevens WJ (2008). Basophil activation test by flow cytometry: present and future applications in allergology. Cytometry B Clin Cytom.

[5] Kleine-Tebbe J, Erdmann S, Knol EF, MacGlashan DW, Poulsen LK, Gibbs BF (2006). Diagnostic tests based on human basophils: potentials, pitfalls and perspectives. Int Arch Allergy Immunol.

[6] Gober LM, Eckman JA, Sterba PM, Vasagar K, Schroeder JT, Golden DB, Saini SS (2007). Expression of activation markers on basophils in a controlled model of anaphylaxis. J Allergy Clin Immunol.

[7] Commins SP, James HR, Stevens W, Pochan SL, Land MH, King C, Mozzicato S, Platts-Mills TA (2014). Delayed clinical and ex vivo response to mammalian meat in patients with IgE to galactose-alpha-1,3-galactose. J Allergy Clin Immunol.

[8] Knol EF, Mul FP, Jansen H, Calafat J, Roos D (1991). Monitoring human basophil activation via CD63 monoclonal antibody 435. J Allergy Clin Immunol.

[9] Hauswirth AW, Natter S, Ghannadan M, Majlesi Y, Schernthaner GH, Sperr WR, Buhring HJ, Valenta R, Valent P (2002). Recombinant allergens promote expression of CD203c on basophils in sensitized individuals. J Allergy Clin Immunol.

[10] Amano T, Furuno T, Hirashima N, Ohyama N, Nakanishi M (2001). Dynamics of intracellular granules with CD63-GFP in rat basophilic leukemia cells. J Biochem.

[11] Hennersdorf F, Florian S, Jakob A, Baumgartner K, Sonneck K, Nordheim A, Biedermann T, Valent P, Buhring HJ (2005). Identification of CD13, CD107a, and CD164 as novel basophil-activation markers and dissection of two response patterns in time kinetics of IgE-dependent upregulation. Cell Res.

[12] Glaumann S, Nopp A, Johansson SG, Rudengren M, Borres MP, Nilsson C (2012). Basophil allergen threshold sensitivity, CD-sens, IgE-sensitization and DBPCFC in peanut-sensitized children. Allergy.

[13] Johansson SG, Nopp A, van Hage M, Olofsson N, Lundahl J, Wehlin L, Soderstrom L, Stiller V, Oman H (2005). Passive IgE-sensitization by blood transfusion. Allergy.

[14] Patil SU, Shreffler WG (2012). Immunology in the Clinic Review Series; focus on allergies: basophils as biomarkers for assessing immune modulation. Clin Exp Immunol.

[15] Santos AF, Douiri A, Becares N, Wu SY, Stephens A, Radulovic S, Chan SM, Fox AT, Du Toit G, Turcanu V (2014). Basophil activation test discriminates between allergy and tolerance in peanut-sensitized children. J Allergy Clin Immunol.

[16] Javaloyes G, Goikoetxea MJ, Garcia Nunez I, Sanz ML, Blanca M, Scheurer S, Vieths S, Ferrer M (2012). Performance of different in vitro techniques in the molecular diagnosis of peanut allergy. J Investig Allergol Clin Immunol.

[17] Ocmant A, Mulier S, Hanssens L, Goldman M, Casimir G, Mascart F, Schandene L (2009). Basophil activation tests for the diagnosis of food allergy in children. Clin Exp Allergy.

[18] Glaumann S, Nopp A, Johansson SG, Borres MP, Nilsson C (2013). Oral peanut challenge identifies an allergy but the peanut allergen threshold sensitivity is not reproducible. PLoS One.

[19] Sato S, Tachimoto H, Shukuya A, Kurosaka N, Yanagida N, Utsunomiya T, Iguchi M, Komata T, Imai T, Tomikawa M (2010). Basophil activation marker CD203c is useful in the diagnosis of hen’s egg and cow’s milk allergies in children. Int Arch Allergy Immunol.

[20] Ciepiela O, Zwiazek J, Zawadzka-Krajewska A, Kotula I, Kulus M, Demkow U (2010). Basophil activation test based on the expression of CD203c in the diagnostics of cow milk allergy in children. Eur J Med Res.

[21] Carroccio A, Brusca I, Mansueto P, D’Alcamo A, Barrale M, Soresi M, Seidita A, La Chiusa SM, Iacono G, Sprini D (2013). A comparison between two different in vitro basophil activation tests for gluten- and cow’s milk protein sensitivity in irritable bowel syndrome (IBS)-like patients. Clin Chem Lab Med.

[22] Tokuda R, Nagao M, Hiraguchi Y, Hosoki K, Matsuda T, Kouno K, Morita E, Fujisawa T (2009). Antigen-induced expression of CD203c on basophils predicts IgE-mediated wheat allergy. Allergol Int.

[23] Chinuki Y, Kaneko S, Dekio I, Takahashi H, Tokuda R, Nagao M, Fujisawa T, Morita E (2012). CD203c expression-based basophil activation test for diagnosis of wheat-dependent exercise-induced anaphylaxis. J Allergy Clin Immunol.

[24] Carroccio A, Mansueto P, Iacono G, Soresi M, D’Alcamo A, Cavataio F, Brusca I, Florena AM, Ambrosiano G, Seidita A (2012). Non-celiac wheat sensitivity diagnosed by double-blind placebo-controlled challenge: exploring a new clinical entity. Am J Gastroenterol.

[25] Carroccio A, Brusca I, Mansueto P, Pirrone G, Barrale M, Di Prima L, Ambrosiano G, Iacono G, Lospalluti ML, La Chiusa SM (2010). A cytologic assay for diagnosis of food hypersensitivity in patients with irritable bowel syndrome. Clin Gastroenterol Hepatol.

[26] Cucu T, De Meulenaer B, Bridts C, Devreese B, Ebo D (2012). Impact of thermal processing and the Maillard reaction on the basophil activation of hazelnut allergic patients. Food Chem Toxicol.

[27] Worm M, Hompes S, Fiedler EM, Illner AK, Zuberbier T, Vieths S (2009). Impact of native, heat-processed and encapsulated hazelnuts on the allergic response in hazelnut-allergic patients. Clin Exp Allergy.

[28] Brandstrom J, Nopp A, Johansson SG, Lilja G, Sundqvist AC, Borres MP, Nilsson C (2015). Basophil allergen threshold sensitivity and component resolved diagnostics improve hazelnut allergy diagnosis. Clin Exp Allergy.

[29] Ebo DG, Bridts CH, Hagendorens MM, De Clerck LS, Stevens WJ (2008). Scampi allergy: from fancy name-giving to correct diagnosis. J Investig Allergol Clin Immunol.

[30] Gamboa PM, Sanz ML, Lombardero M, Barber D, Sanchez-Monje R, Goikoetxea MJ, Antepara I, Ferrer M, Salcedo G (2009). Component-resolved in vitro diagnosis in peach-allergic patients. J Investig Allergol Clin Immunol.

[31] Gamboa PM, Caceres O, Antepara I, Sanchez-Monge R, Ahrazem O, Salcedo G, Barber D, Lombardero M, Sanz ML (2007). Two different profiles of peach allergy in the north of Spain. Allergy.

[32] Diaz-Perales A, Sanz ML, Garcia-Casado G, Sanchez-Monge R, Garcia-Selles FJ, Lombardero M, Polo F, Gamboa PM, Barber D, Salcedo G (2003). Recombinant Pru p 3 and natural Pru p 3, a major peach allergen, show equivalent immunologic reactivity: a new tool for the diagnosis of fruit allergy. J Allergy Clin Immunol.

[33] Erdmann SM, Heussen N, Moll-Slodowy S, Merk HF, Sachs B (2003). CD63 expression on basophils as a tool for the diagnosis of pollen-associated food allergy: sensitivity and specificity. Clin Exp Allergy.

[34] Ebo DG, Hagendorens MM, Bridts CH, Schuerwegh AJ, De Clerck LS, Stevens WJ (2005). Flow cytometric analysis of in vitro activated basophils, specific IgE and skin tests in the diagnosis of pollen-associated food allergy. Cytometry B Clin Cytom.

[35] Erdmann SM, Sachs B, Schmidt A, Merk HF, Scheiner O, Moll-Slodowy S, Sauer I, Kwiecien R, Maderegger B, Hoffmann-Sommergruber K (2005). In vitro analysis of birch-pollen-associated food allergy by use of recombinant allergens in the basophil activation test. Int Arch Allergy Immunol.

[36] Raap U, Wieczorek D, Schenck F, Kapp A, Wedi B (2011). The basophil activation test is a helpful diagnostic tool in anaphylaxis to sesame with false-negative specific IgE and negative skin test. Allergy.

[37] Trcka J, Schad SG, Scheurer S, Conti A, Vieths S, Gross G, Trautmann A (2012). Rice-induced anaphylaxis: IgE-mediated allergy against a 56-kDa glycoprotein. Int Arch Allergy Immunol.

[38] Chiang WC, Huang CH, Llanora GV, Gerez I, Goh SH, Shek LP, Nauta AJ, Van Doorn WA, Bindels J, Ulfman LH (2012). Anaphylaxis to cow’s milk formula containing short-chain galacto-oligosaccharide. J Allergy Clin Immunol.

[39] Hoffmann HJ, Santos AF, Mayorga C, Nopp A, Eberlein B, Ferrer M, Rouzaire P, Ebo DG, Sabato V, Sanz ML (2015). The clinical utility of basophil activation testing in diagnosis and monitoring of allergic disease. Allergy.

[40] Santos AF, Du Toit G, Lack G (2015). Is the use of epinephrine a good marker of severity of allergic reactions during oral food challenges?. J Allergy Clin Immunol Pract.

[41] Chirumbolo S, Vella A, Ortolani R, De Gironcoli M, Solero P, Tridente G, Bellavite P (2008). Differential response of human basophil activation markers: a multi-parameter flow cytometry approach. Clin Mol Allergy.

[42] Hausmann OV, Gentinetta T, Fux M, Ducrest S, Pichler WJ, Dahinden CA (2011). Robust expression of CCR3 as a single basophil selection marker in flow cytometry. Allergy.

[43] Santos AF, Becares N, Stephens A, Turcanu V, Lack G (2016). The expression of CD123 can decrease with basophil activation – implications for the gating strategy of the basophil activation test. Clinical and Translational Allergy..

[44] Rubio A, Vivinus-Nebot M, Bourrier T, Saggio B, Albertini M, Bernard A (2011). Benefit of the basophil activation test in deciding when to reintroduce cow’s milk in allergic children. Allergy.

[45] Nilsson N, Nilsson C, Hedlin G, Johansson SG, Borres MP, Nopp A (2013). Combining Analyses of Basophil Allergen Threshold Sensitivity, CD-sens, and IgE Antibodies to Hydrolyzed Wheat, omega-5 Gliadin and Timothy Grass Enhances the Prediction of Wheat Challenge Outcome. Int Arch Allergy Immunol.

[46] Santos AF, Du Toit G, Douiri A, Radulovic S, Stephens A, Turcanu V, Lack G (2015). Distinct parameters of the basophil activation test reflect the severity and threshold of allergic reactions to peanut. J Allergy Clin Immunol.

[47] Song Y, Wang J, Leung N, Wang LX, Lisann L, Sicherer SH, Scurlock AM, Pesek R, Perry TT, Jones SM (2015). Correlations between basophil activation, allergen-specific IgE with outcome and severity of oral food challenges. Ann Allergy Asthma Immunol.

[48] Wanich N, Nowak-Wegrzyn A, Sampson HA, Shreffler WG (2009). Allergen-specific basophil suppression associated with clinical tolerance in patients with milk allergy. J Allergy Clin Immunol.

[49] Ford LS, Bloom KA, Nowak-Wegrzyn AH, Shreffler WG, Masilamani M, Sampson HA: Basophil reactivity, wheal size, and immunoglobulin levels distinguish degrees of cow’s milk tolerance. J Allergy Clin Immunol. 2012([epub ahead of print]). 10.1016/j.jaci.2012.06.003 PMC3493710 22819512

[50] Wood RA, Sicherer SH, Burks AW, Grishin A, Henning AK, Lindblad R, Stablein D, Sampson HA (2013). A phase 1 study of heat/phenol-killed, *E. coli*-encapsulated, recombinant modified peanut proteins Ara h 1, Ara h 2, and Ara h 3 (EMP-123) for the treatment of peanut allergy. Allergy.

[51] Fleischer DM, Burks AW, Vickery BP, Scurlock AM, Wood RA, Jones SM, Sicherer SH, Liu AH, Stablein D, Henning AK (2013). Sublingual immunotherapy for peanut allergy: a randomized, double-blind, placebo-controlled multicenter trial. J Allergy Clin Immunol.

[52] Thyagarajan A, Jones SM, Calatroni A, Pons L, Kulis M, Woo CS, Kamalakannan M, Vickery BP, Scurlock AM, Wesley Burks A (2012). Evidence of pathway-specific basophil anergy induced by peanut oral immunotherapy in peanut-allergic children. Clin Exp Allergy.

[53] Kim EH, Bird JA, Kulis M, Laubach S, Pons L, Shreffler W, Steele P, Kamilaris J, Vickery B, Burks AW (2011). Sublingual immunotherapy for peanut allergy: clinical and immunologic evidence of desensitization. J Allergy Clin Immunol.

[54] Jones SM, Pons L, Roberts JL, Scurlock AM, Perry TT, Kulis M, Shreffler WG, Steele P, Henry KA, Adair M (2009). Clinical efficacy and immune regulation with peanut oral immunotherapy. J Allergy Clin Immunol.

[55] Keet CA, Frischmeyer-Guerrerio PA, Thyagarajan A, Schroeder JT, Hamilton RG, Boden S, Steele P, Driggers S, Burks AW, Wood RA (2012). The safety and efficacy of sublingual and oral immunotherapy for milk allergy. J Allergy Clin Immunol.

[56] Burks AW, Jones SM, Wood RA, Fleischer DM, Sicherer SH, Lindblad RW, Stablein D, Henning AK, Vickery BP, Liu AH (2012). Oral immunotherapy for treatment of egg allergy in children. N Engl J Med.

[57] Vila L, Moreno A, Gamboa PM, Martinez-Aranguren R, Sanz ML (2013). Decrease in antigen-specific CD63 basophil expression is associated with the development of tolerance to egg by SOTI in children. Pediatr Allergy Immunol.

[58] Gernez Y, Tirouvanziam R, Yu G, Ghosn EE, Reshamwala N, Nguyen T, Tsai M, Galli SJ, Herzenberg LA, Nadeau KC (2011). Basophil CD203c levels are increased at baseline and can be used to monitor omalizumab treatment in subjects with nut allergy. Int Arch Allergy Immunol.

[59] Patil SP, Wang J, Song Y, Noone S, Yang N, Wallenstein S, Sampson HA, Li XM (2011). Clinical safety of Food Allergy Herbal Formula-2 (FAHF-2) and inhibitory effect on basophils from patients with food allergy: Extended phase I study. J Allergy Clin Immunol.

